# Historical distribution and host-vector diversity of *Francisella tularensis,* the causative agent of tularemia, in Ukraine

**DOI:** 10.1186/s13071-014-0453-2

**Published:** 2014-10-16

**Authors:** Jake Hightower, Ian T Kracalik, Nataliya Vydayko, Douglas Goodin, Gregory Glass, Jason K Blackburn

**Affiliations:** Department of Geography, Spatial Epidemiology & Ecology Research Laboratory, University of Florida, Gainesville, FL USA; Emerging Pathogens Institute, University of Florida, Gainesville, FL USA; Central Sanitary Epidemiological Station, Kyiv, Ukraine; Department of Geography, Kansas State University, Manhattan, KS USA; Johns Hopkins Bloomberg School of Public Health, Johns Hopkins University, Baltimore, MD USA

**Keywords:** Tularemia, SaTScan, Spatial clusters, Ukraine, Land cover, *Francisella tularensis*

## Abstract

**Background:**

*Francisella tularensis,* the causative agent of tularemia, is a zoonotic agent that remains across much of the northern hemisphere, where it exists in enzootic cycles. In Ukraine, tularemia has a long history that suggests a need for sustained surveillance in natural foci. To better characterize the host-vector diversity and spatial distribution of tularemia, we analyzed historical data from field collections carried out from 1941 to 2008.

**Findings:**

We analyzed the spatial-temporal distribution of bacterial isolates collected from field samples. Isolates were characterized by source and dominant land cover type. To identify environmental persistence and spatial variation in the source of isolation, we used the space-time permutation and multinomial models in SaTScan. A total of 3,086 positive isolates were taken from 1,084 geographic locations. Isolation of *F. tularensis* was more frequent among arthropods [n = 2,045 (66.3%)] followed by mammals [n = 619 (20.1%)], water [n = 393 (12.7%)], and farm produce [n = 29 (0.94%)], respectively. Four areas of persistent bacterial isolation were identified. Water and farm produce as sources of bacterial isolation were clustered.

**Conclusions:**

Our findings confirm the presence of long-standing natural foci of *F. tularensis* in Ukraine. Given the history of tularemia as well as its environmental persistence there exists a possibility of (re)emergence in human populations. Heterogeneity in the distribution of tularemia isolate recovery related to land cover type supports the theory of natural nidality and clusters identify areas to target potential sources of the pathogen and improve surveillance.

**Electronic supplementary material:**

The online version of this article (doi:10.1186/s13071-014-0453-2) contains supplementary material, which is available to authorized users.

## Findings

*Francisella tularensis,* the causative agent of tularemia, is a zoonotic, gram-negative bacterium that is broadly distributed across the Northern Hemisphere, with most human cases caused by either *F. tularensis tularensis or F.t. holarctica,* with cases from the latter less severe [1]. Generally, *F.t. holarctica* is most common across Europe, though *F.t. tularensis* has been recovered in central Europe [2]. Human exposure may occur through various pathways including arthropod bites, ingesting contaminated food products or liquids, inhaling aerosolized bacteria, or handling infected animals [1].

Despite a global decline in reported human cases [3], tularemia has recently (re)emerged in several countries including Sweden, Kosovo, China, Bulgaria [4] and parts of the former Soviet Union (FSU) [5]. Historically, outbreaks in the FSU were linked to small mammals and arthropods (ticks), possibly related to increases in host or vector population abundance [6,7]. More recent waterborne outbreaks of tularemia in Bulgaria [8] and Georgia [9], have reinforced the need for continued surveillance and preventative measures in endemic areas. In Ukraine, the first confirmed cases of tularemia were documented in the 1940's [4,10]. Those cases were associated with occupational exposure in furriers, whereas subsequent outbreaks were linked to rodent populations attracted to sugar factory production [4,10]. Tularemia foci were previously described in the 1960's across a limited geography in the south of Ukraine where several arthropods and small mammals were recognized as competent vectors and hosts [4,11]. However, contemporary characterizations of the spatial distribution and composition of vectors and hosts are incomplete and should be further analyzed.

Active disease surveillance is expensive and labor intensive. The use of historical data can direct such efforts for more efficient allocation of resources. Recent studies have shown that incorporating historical data and spatial analysis can improve vector-borne and zoonotic disease surveillance [12-15]. This study describes the spatial-temporal characteristics of tularemia across Ukraine, as well variation in its hosts and vectors to better understand its distribution, epidemiology, and improve surveillance.

From 1941 to 2008, the Central Sanitary Epidemiological Station (CSES) in Ukraine collected field samples nationally to test for *F. tularensis*. Confirmation of *F. tularensis* was performed via traditional bacterial culturing techniques [16]*.* This isolate collection included positive cultures from mammals, arthropods, and environmental sources, as well as ancillary information on the date and location of sample collections. We mapped *F. tularensis* positive isolates by year and by source of bacterial recovery. For this study, the historical database did not differentiate subspecies so we refer broadly to isolates as *F. tularensis*. Land cover (LC) characteristics were derived from the contemporary Globcover LC dataset [17] and assigned to each isolate. GlobCover data were reclassified into five broad categories: rain fed croplands, mosaic croplands, broadleaved forest, grass and shrub lands, and other (water, bare areas, and urban) (Additional file 1: Figure S1). Mapping was performed using ArcGIS v10.1 (ESRI, Redlands, CA).

We tested for space-time clustering of *F. tularensis* isolates using the retrospective space-time scan statistic in SaTScan™ v9.0 [18] with the space-time permutation model. Significant clusters represented geographic tularemia foci (defined as persistent bacterial isolation over periods >1 year). The space-time permutation model was most appropriate as the isolate database only recorded isolates and not the total field collection effort per isolate. The space-time permutation is described in detail elsewhere [19]. Briefly, the test creates multiple varying sized ‘cylinders’ around each case, where the circular base represents space and the cylinder height represents time. To determine if presence-only data are clustered in space and time, the number of cases in a cylinder is compared to case expectations outside of the cylinder. The model was run using year of isolation with the maximum spatial and temporal windows set to 50% of the population and 50% of the study period. Statistical significance of clusters was evaluated through Monte Carlo simulations, generating 999 random permutations to obtain p-values and selecting clusters with p-values ≤ 0.05, which were overlain with historical foci documented in Pollitzer [20]. A second categorical multinomial model was run in SaTScan, using the aforementioned parameter settings, to test for the presence of space-time clustering among isolate sources grouped as: mammals, arthropods, farm produce, and water. This test identifies geographic areas (here isolate locations) with an increased occurrence of a particular isolate source. To test for differences in LC characteristics, we calculated the proportion of isolates identified as space-time clusters (within foci) and non-clusters within each of the five Globcover-defined LC classes were compared using a chi-square analysis.

**Figure 1:**
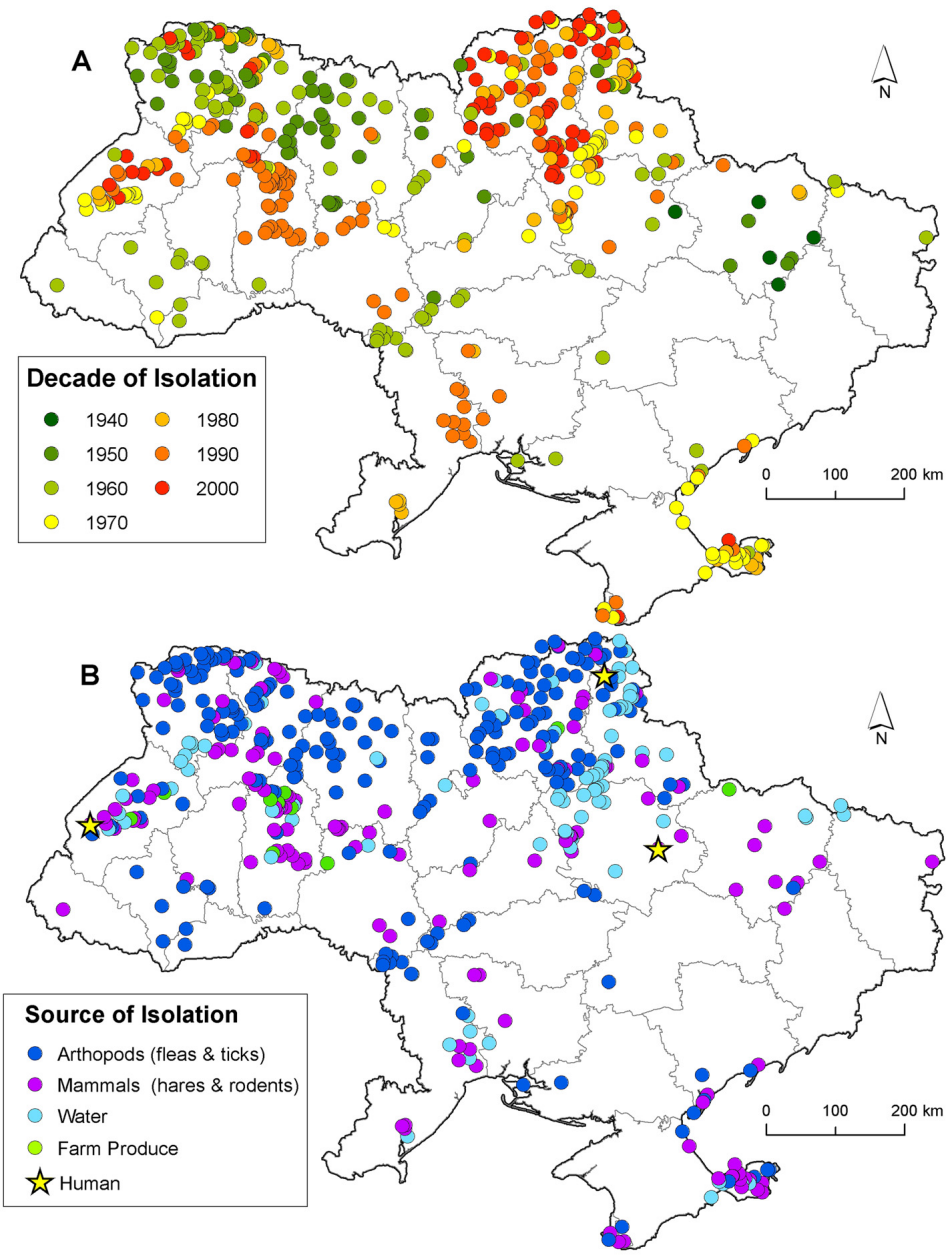
**Map inset A shows the distribution of** ***Francisella tularensis*** **isolates collected by decade.** Map inset **B** shows the distribution of isolates by source.

**Figure 2:**
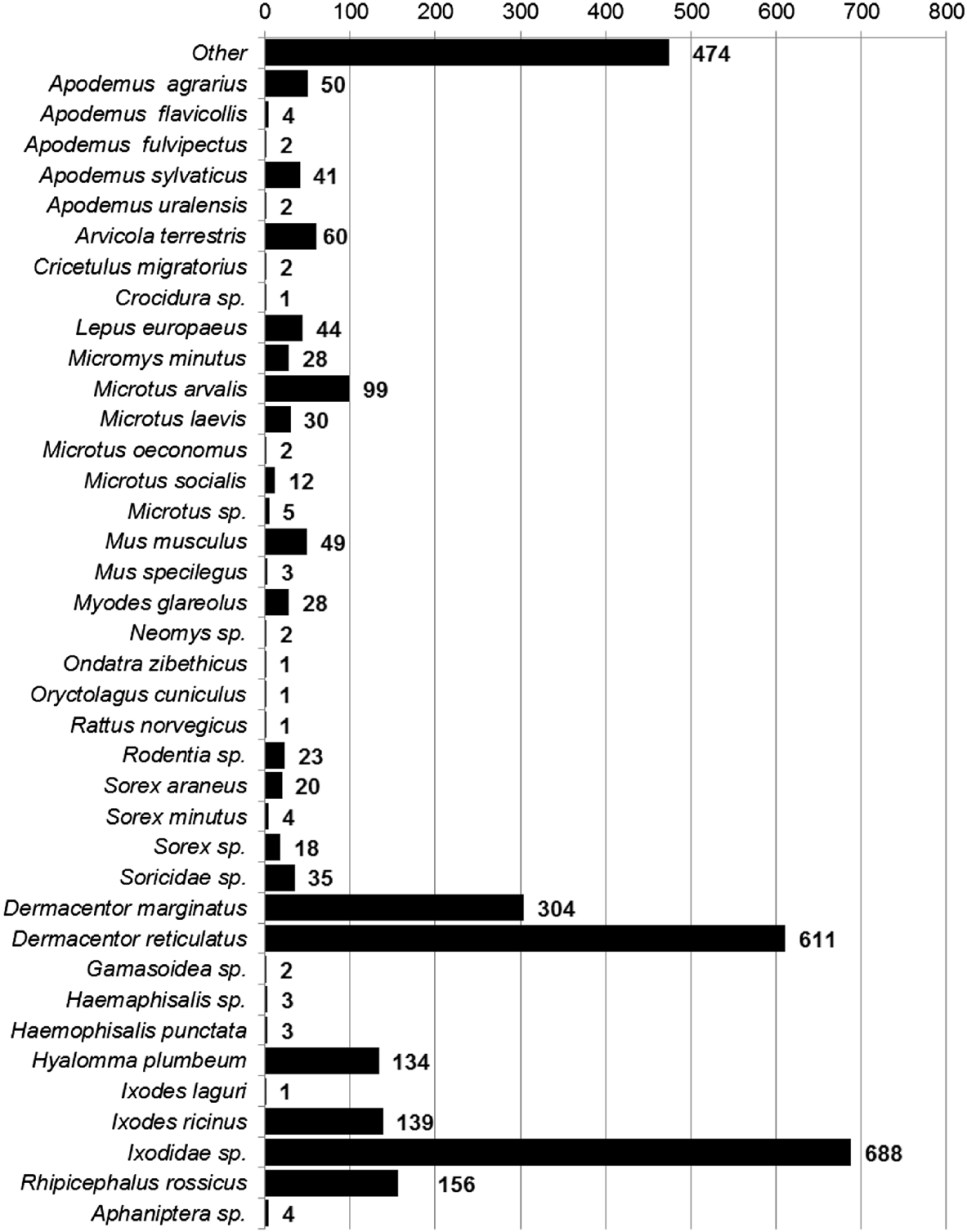
**Source of** ***Francisella tularensis*** **isolation by genus and species.**

From 1941-2008, 3,086 positive isolates of *F. tularensis* were recovered from samples collected at 1,084 locations. Figure 1 illustrates the spatial distribution of isolates by decade and source of isolation. The fewest number of isolates were obtained during the 1940's (n = 18). The greatest number were recorded during the 1970's (n = 840). Arthropods were the most common source [n = 2,045 (66.3%)] followed by mammals [n = 619 (20.1%)], water [n = 393 (12.7%)], and farm produce [n = 29 (0.94%),] (Additional file 1: Figure S1). The number of isolates per taxa are shown in Figure 2. *F. tularensis* was most frequently isolated from *Dermacentor spp.* ticks (29.7%) and *Microtus spp.* rodents (4.8%), respectively. LC characteristics of the isolates showed a large number were collected within rain-fed croplands [n = 542 (49.1%)], with category 'other' comprising the highest percentage in relation to total available LC (Additional file 2: Figure S2).

**Figure 3:**
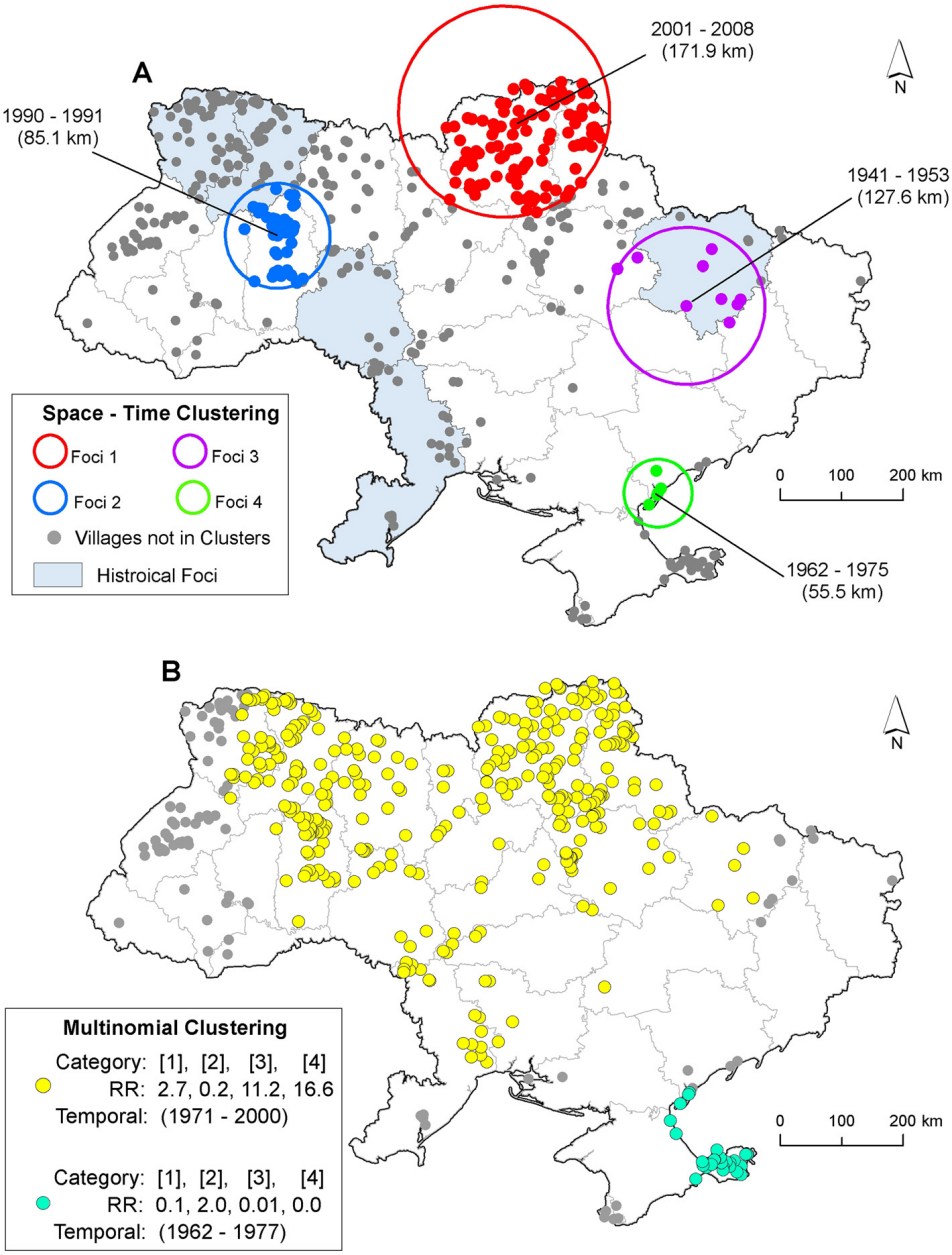
**Spatial clusters of** ***Francisella tularensis*** **isolates in Ukraine.** Map inset **A** displays the results of the SaTScan space-time analysis of all *Francisella tularensis* isolates identifying foci of isolation. Results are overlain with Historical foci documented in Pollitzer 1967 [18]. Circles represent the spatial extent of a given cluster in kilometers (km). Time periods indicate the duration of the cluster in years. Map inset **B** displays the results of the multinomial cluster model. Categories in brackets refer to the source of isolation: 1 = mammals, 2 = arthropods, 3 = water, and 4 = farm produce. Relative risk (RR) estimates below the brackets indicate whether or not greater than expected number of isolations occurred in a given category. RR >1 represent a greater than expected number of isolations.

Four tularemia foci (areas of persistent bacterial isolation) were identified by the space-time statistic (Table 1). Foci ranged in duration two years to fourteen years (Figure 3A). The proportion of isolates within the SaTScan defined foci across five LC classes was significantly different from the proportion of isolates within non-foci (χ^2^ = 48.72, DF = 4, p < 0.001). The Multinomial SatScan model showed that during the period 1971-2000, there was greater occurrence of isolation among the water and farm produce categories, displayed in yellow, whereas during the earlier period 1962-1977 an isolation among arthropods was higher, displayed in light blue (Figure 3B).

**Table 1:** **Results of the SaTScan space-time permutation analyses identifying foci of** ***Francisella tularensis*** **that persisted > 1 year**

**Cluster (Foci)**	**Isolates**	**Expected**	**Test Statistic**	**p-Value**
**1**	217	32.69	232.12	0.001
**2**	105	8.32	171.09	0.001
**3**	999	571.9	168.66	0.001
**4**	26	0.45	79.82	0.001

Our findings support evidence of long-standing natural foci of *F. tularensis* in Ukraine. Heterogeneity in the recovery of bacterial isolates by geographic location support the theory of natural nidality [21]. Our findings confirmed observations from the 1960's that documented foci in western Ukraine [20]. We also identified foci in the southeast of the country and elucidated areas of bacterial isolation that persisted through last decade (2000's). The distribution of foci, vectors, hosts, and infectious agents are known to be influenced by ecological characteristics [22]; consistent with this association, our analysis indicated differences in LC among areas identified as foci and non-foci.

Foci in the FSU were historically classified by ecotypes/LC (steppe, forest, foothills) with the most ubiquitous type consisting of flood/marshlands [20,23], associated with areas of sugar beet production and mixed agriculture use [5]. In keeping with previous research we noted that the recovery of *F. tularensis* in Ukraine occurred primarily in croplands followed by forests, shrub/grasslands (steppe), and water. Interestingly, we found variation in the space-time distribution of the sources of bacterial recovery indicating a greater than expected number of isolates in mammals, water, and farm produce during 1971-2000 (Figure 3B). Farm produce and water can become contaminated by infected mammals, particularly semi-aquatic rodents (*Arvicola terrestris*)*,* which have been linked to human outbreaks from agriculture and sugar factories [5,20]. This cluster may have indicated an increased abundance in rodents or a more concerted sampling effort brought on by concern over water-borne outbreaks in humans, often the source of epidemics [7-9,24]. Additionally, a cluster of arthropod isolates during 1962-1977 was identified in the southeast where, in some areas, the vector *D. marginatus* was historically shown to have bacterial recovery rates of 2.5% and played an important role in human transmission and maintaining the enzootic cycle of the pathogen [25]. This cluster is in contrast to the ecotype in western Ukraine, historically classified as steppe [5,20,23] and supports the hypotheses of geographic variation in the epidemiology of human tularemia [5,20,25,26].

Differences in the sampling efforts conducted year to year may partly account for the patterns identified here. Although recovery rates were not available for our data, we incorporated the SaTScan methodology to successfully identify foci and persistence of bacterial isolation due its flexibility in dealing with missing data. Despite the lack of historical human case data, sampling efforts may have been driven by human outbreaks, as were previously documented across Ukraine [25]. High resolution human disease data from the FSU are extremely difficult to obtain. This fact coupled with mass vaccination campaigns and the presence of the less virulent *F. tularensis holarcitica* likely contributed to an underestimation in reporting [5,27]. While bacterial recovery rates were not available for the entire data set, limited field collections from the 1950's and 60's showed recovery of *F. tularensis* was 0.2% (265/199,343) from the ticks collected [25]. The contemporary GlobCover database may have misclassified LC characteristics of historical isolates. As a first effort, we calculated the percent change in cropland by decade from 1940 – 2000 using HYDE [28] (Additional file 3; Additional file 4: Figure S3). Overall, there was a slight increase in cropland across the decades.

Disease surveillance is costly and time consuming especially when monitoring is conducted with limited empirical or historical data to identify priority areas. Given the challenges faced by newly independent FSU states, limited public health resources make such monitoring infeasible. This study showed that archived, historical records provide important clues to identify important vectors, hosts, environmental sources, and geographic foci for *F. tularensis*. The persistence of environmental foci in Ukraine gives rise to the possibility of (re)emergence in human populations. Our findings can be used to inform more efficient targeted surveillance strategies and monitoring of tularemia foci. The recurrent isolation of *F. tularensis* from several sources (wild rodents, croplands and water) points to the need for continued surveillance. The LC analysis suggests that areas of mixed agriculture (croplands) should be prioritized as areas for future surveillance, with an emphasis on rodent and tick species defined here.

## Additional files

Additional file 1: Figure S1. Land cover categories in Ukraine based on the GlobCover dataset and reclassified into five categories: rain-fed croplands, mosaic croplands, broadleaved forests, grass/shrub lands, and other. Additional file 2: Figure S2. The top panel shows the proportion of the *Francisella tularensis* isolates that fell within each of the five Land cover classes. The bottom panel shows the percentage of the LC in Ukraine by category. Additional file 3:
**Supplemental information.**
Additional file 4: Figure S3. Graph illustrates the total area of cropland Land cover (LC) type in Ukraine by decade based on the HYDE historical database. Percent values above bars represent the change in cropland LC for each decade using 2000 as the reference.

## Abbreviations

CSES: Central sanitary epidemiological station
GIS: Geographic information systems
FSU: Former soviet union
LC: Land cover
