# Supplementary figures and images for: Historical distribution and host-vector diversity of Francisella tularensis, the causative agent of tularemia, in Ukraine
Source: Parasit Vectors. 2014 Oct 16;7:453. doi: 10.1186/s13071-014-0453-2 (PMC4200231; doi:10.1186/s13071-014-0453-2)

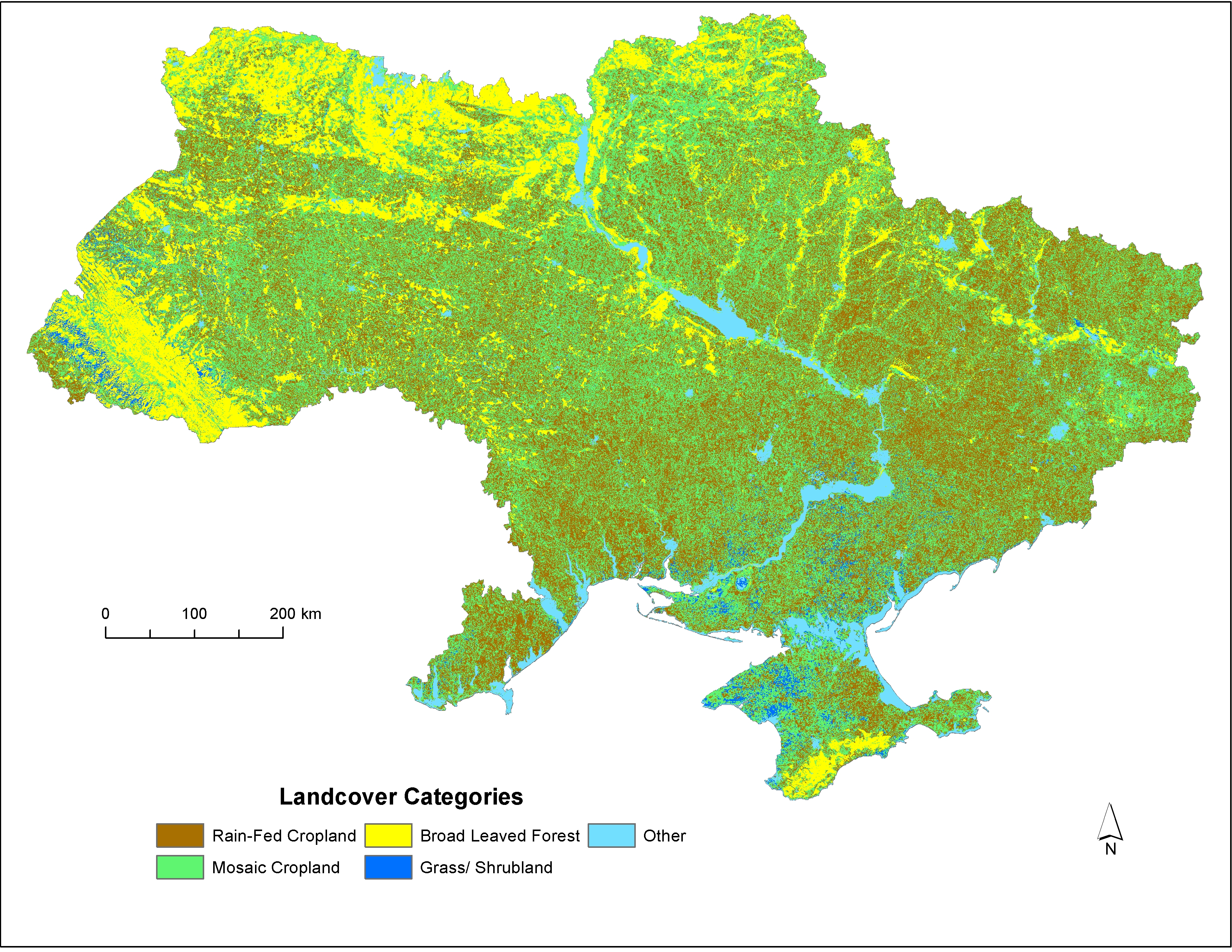

Supplement: Additional file 1: Figure S1. — Land cover categories in Ukraine based on the GlobCover dataset and reclassified into five categories: rain-fed croplands, mosaic croplands, broadleaved forests, grass/shrub lands, and other. [file 13071_2014_453_MOESM1_ESM.jpeg]

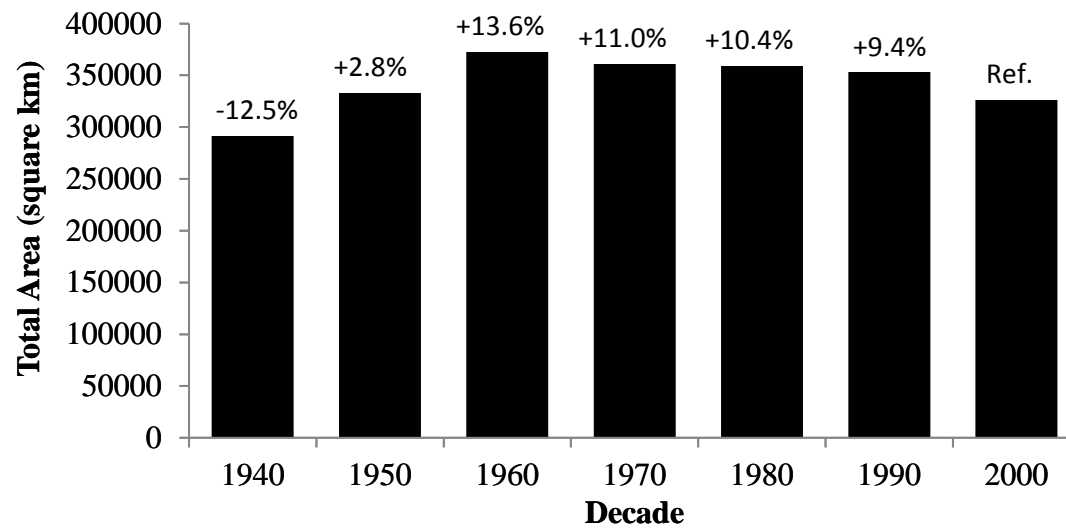

Supplement: Additional file 4: Figure S3. — Graph illustrates the total area of cropland Land cover (LC) type in Ukraine by decade based on the HYDE historical database. Percent values above bars represent the change in cropland LC for each decade using 2000 as the reference. [file 13071_2014_453_MOESM4_ESM.pdf]
