# Supplementary material for: Historical distribution and host-vector diversity of Francisella tularensis, the causative agent of tularemia, in Ukraine
Source: Parasit Vectors. 2014 Oct 16;7:453. doi: 10.1186/s13071-014-0453-2 (PMC4200231; doi:10.1186/s13071-014-0453-2)
Supplement: Additional file 2: Figure S2. — The top panel shows the proportion of the Francisella tularensis isolates that fell within each of the five Land cover classes. The bottom panel shows the percentage of the LC in Ukraine by category. [file 13071_2014_453_MOESM2_ESM.pptx]

## Slide 1
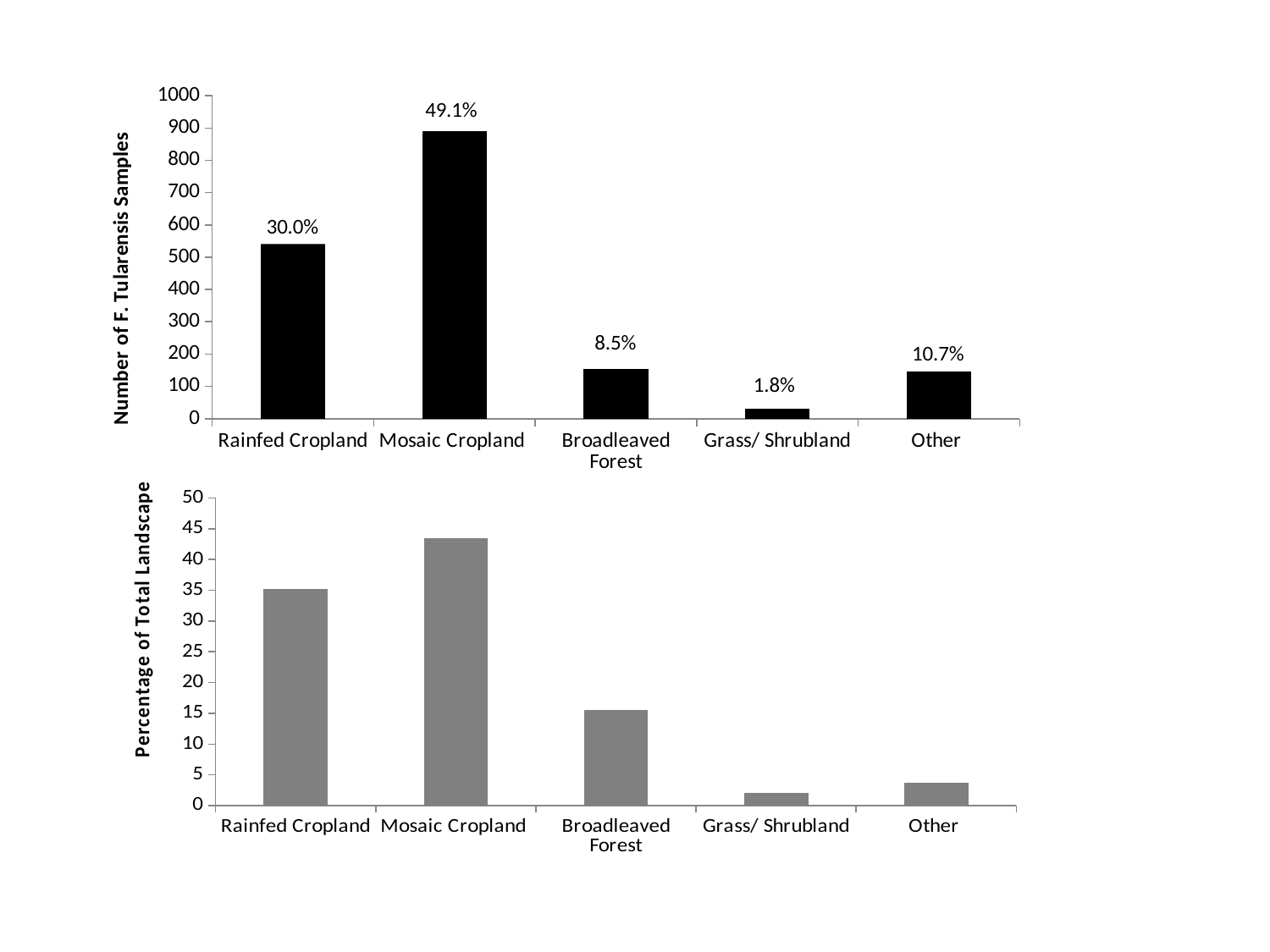

### Chart
| Category | |
|---|---|
| Rainfed Cropland | 542.0 |
| Mosaic Cropland | 889.0 |
| Broadleaved Forest | 153.0 |
| Grass/ Shrubland | 32.0 |
| Other | 193.0 |
### Chart
| Category | |
|---|---|
| Rainfed Cropland | 35.2 |
| Mosaic Cropland | 43.4 |
| Broadleaved Forest | 15.6 |
| Grass/ Shrubland | 2.1 |
| Other | 3.7 |

## Slide 2
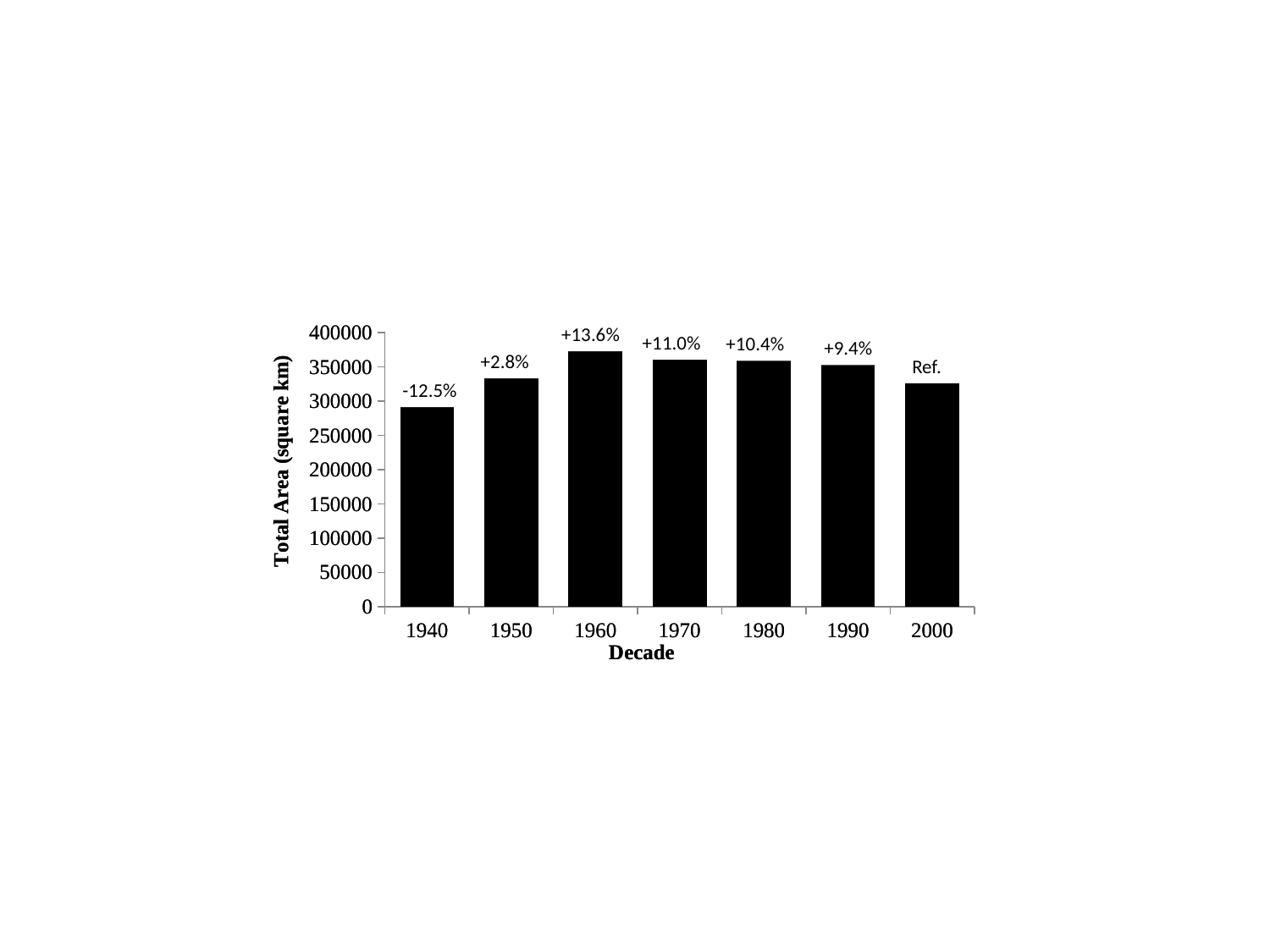

### Chart
| Category | Toal Area of Cropland (km2) |
|---|---|
| 1940 | 292194.0 |
| 1950 | 338379.0 |
| 1960 | 380404.0 |
| 1970 | 369437.0 |
| 1980 | 367028.0 |
| 1990 | 362840.0 |
| 2000 | 328799.0 |
### Chart
| Category | Toal Area of Cropland (km2) |
|---|---|
| 1940 | 292194.0 |
| 1950 | 338379.0 |
| 1960 | 380404.0 |
| 1970 | 369437.0 |
| 1980 | 367028.0 |
| 1990 | 362840.0 |
| 2000 | 328799.0 |+13.6%
+11.0%
+10.4%
+9.4%
+2.8%
Ref.
-12.5%
