# Supplementary material for: Historical distribution and host-vector diversity of Francisella tularensis, the causative agent of tularemia, in Ukraine
Source: Parasit Vectors. 2014 Oct 16;7:453. doi: 10.1186/s13071-014-0453-2 (PMC4200231; doi:10.1186/s13071-014-0453-2)
Supplement: Additional file 3: — Supplemental information. [file 13071_2014_453_MOESM3_ESM.pdf]

**Supplemental Information**

Land cover may change over time due to economic, population, or agricultural drivers. In our analysis, estimates of land cover type for the bacterial isolates may have been biased due to the fact that GlobCover [1] is based on year 2000 land cover estimates and our samples date back to the 1940's. We used the HYDE historical land cover database [2], which contains decadal estimates of cropland dating back to 10,000 B.C. at ~ 5' by 5' spatial resolution, to determine the amount of change in cropland that has taken place during our study period (1941 through 2008). We calculated the total area of cropland in Ukraine for each decade (1940 to 2000). The percent change in cropland land cover type was then estimated for each decade using 2000 as the point of reference.


1. Arino O, Gross D, Ranera F, Bourg L, Leroy M, Bicheron P, Latham J, Di Gregorio A, Brockman C, Witt R: **GlobCover: ESA service for global land cover from MERIS**. In: *Geoscience and Remote Sensing Symposium, 2007 IGARSS 2007 IEEE International2007*: IEEE: 2412-2415.
2. Goldewijk KK: **Estimating global land use change over the past 300 years: the HYDE database**. *Global Biogeochemical Cycles* 2001, **15**(2):417-433.
